# Protein tyrosine phosphatase PTP1B is a positive regulator of the intracellular development of *Chlamydia trachomatis*

**DOI:** 10.1128/iai.00373-25

**Published:** 2025-10-20

**Authors:** M. Soban Khan, Rom Peles, Anna Haralampiev, Nicholas Becerra, Travis J. Jewett

**Affiliations:** 1 Division of Immunity and Pathogenesis, Burnett School of Biomedical Sciences, College of Medicine, University of Central Florida 6243, Orlando, Florida, USA; University of Pennsylvania Perelman School of Medicine, Philadelphia, Pennsylvania, USA

**Keywords:** inclusions, development, *Chlamydia*, phosphatases

## Abstract

The intracellular survival and replication of *Chlamydia trachomatis* rely on the precise manipulation of host signaling pathways. Host kinases are instrumental in the modulation of host signaling during *C. trachomatis* infection. However, the potential contribution of host phosphatases to chlamydial pathogenesis remains poorly understood. Here, we identified the host tyrosine phosphatase PTP1B as a positive regulator of *C. trachomatis* intracellular development. Gain-of-function approaches revealed that PTP1B promotes inclusion development and increases the production of infectious elementary bodies, whereas loss-of-function by chemical inhibition or silencing leads to a reduction in both inclusion size and bacterial infectivity. Interestingly, PTP1B inhibition did not affect *Chlamydia trachomatis* invasion efficiency, suggesting a specific role during the developmental phase of the chlamydial life cycle. To explore the functional relevance of PTP1B and its potential interaction with chlamydial effectors, we focused on the early-secreted effector Tarp, which undergoes tyrosine phosphorylation upon host cell entry. *In vitro* biochemical assays demonstrated that recombinant PTP1B can dephosphorylate both native and recombinant forms of Tarp. However, PTP1B inhibition during infection did not significantly alter Tarp phosphorylation levels, possibly owing to the overpowering influence of host tyrosine kinases. These findings suggest that while Tarp may not be a major physiological substrate, PTP1B is capable of interacting with phosphorylated chlamydial effectors. Together, these results establish PTP1B as a host factor that supports chlamydial development and underscore the underappreciated role of host phosphatases in bacterial pathogenesis. This study provides a foundation for future work exploring phosphatase-mediated regulation of infection and potential host-directed therapeutic strategies.

## INTRODUCTION

*Chlamydia trachomatis* is the leading cause of bacterial sexually transmitted infections globally and a major contributor to trachoma, the primary infectious cause of preventable blindness in many developing countries (1, 2). This obligate intracellular pathogen undergoes a unique biphasic developmental cycle that alternates between two distinct forms: the infectious elementary body (EB) and the replicative reticulate body (RB) (3). Upon entering host epithelial cells, EBs transition into RBs within a membrane-bound vacuole known as the inclusion, where they replicate (4). To establish and maintain this specialized intracellular niche, *C. trachomatis* extensively manipulates host cellular machinery, including signaling pathways, membrane trafficking systems, cytoskeletal structures, and metabolic networks (5–9). Its capacity to evade immune responses and persist within host cells contributes to chronic infections that can lead to reproductive complications and inflammatory disease (10–12).

Manipulation of the host cell is largely orchestrated by a suite of chlamydial proteins delivered into the host cytosol or to the inclusion membrane via the bacterial type III secretion system (T3SS) (13–15). These bacterial effectors modulate diverse host pathways and are frequently subject to post-translational modifications, particularly phosphorylation by host kinases (16–18). An early effector, the translocated actin-recruiting phosphoprotein (Tarp), is rapidly phosphorylated upon delivery by host Src family kinases and facilitates actin cytoskeletal rearrangements at the site of *Chlamydia* invasion (19, 20). While considerable attention has been devoted to the role of host kinases in supporting effector function and promoting infection (18, 21, 22), the role of host phosphatases, key negative regulators of phosphorylation signaling, remains largely unexplored.

Previous studies have shown that bacterial phosphatases contribute to chlamydial development. For instance, a chlamydial-encoded phosphatase was found to be essential for maintaining inclusion membrane integrity and promoting bacterial growth, underscoring the importance of phosphatase activity for physiology (23). However, the roles of host phosphatases in the context of *C. trachomatis* infection remain poorly understood. This is particularly noteworthy given the dynamic nature of host phosphorylation networks during *C. trachomatis* infection and the potential for phosphatases to fine-tune key signaling events that influence pathogen survival.

The protein tyrosine phosphatase 1B (PTP1B) is a central tyrosine-specific phosphatase that regulates diverse signaling cascades, including those involved in metabolism, immune responses, and vesicle trafficking (24–27). Notably, several chlamydial effectors, including Tarp, undergo tyrosine phosphorylation, highlighting the potential relevance of a tyrosine phosphatase like PTP1B in modulating *C. trachomatis* effector activity during infection. PTP1B is anchored to the cytoplasmic face of the endoplasmic reticulum, enabling it to access both cytosolic and membrane-bound substrates (28). Although understanding of its involvement in infection is limited, PTP1B has been shown to influence host responses during *Pseudomonas aeruginosa* infection (29, 30). Despite this, the contribution of PTP1B to chlamydial infection has not been previously investigated.

In this study, we aimed to investigate whether PTP1B contributes to the intracellular development of *C. trachomatis*. Using pharmacological inhibition, siRNA-mediated knockdown, and overexpression approaches, we found that PTP1B activity directly correlates with inclusion maturation and the generation of infectious progeny. *In vitro* assays demonstrated that PTP1B can dephosphorylate the chlamydial effector, Tarp. However, inhibition of PTP1B during infection did not significantly alter Tarp phosphorylation patterns. These findings demonstrate that PTP1B plays a functional role in supporting chlamydial intracellular development and establish it as an important host enzyme in chlamydial infections. It also provides a foundation for future studies to delineate the downstream host pathways influenced by PTP1B during infection, which may offer new avenues for therapeutic targeting of *C. trachomatis*.

## RESULTS

### Chemical inhibition of the tyrosine phosphatase PTP1B impairs *Chlamydia trachomatis* inclusion development

**Fig 1:**
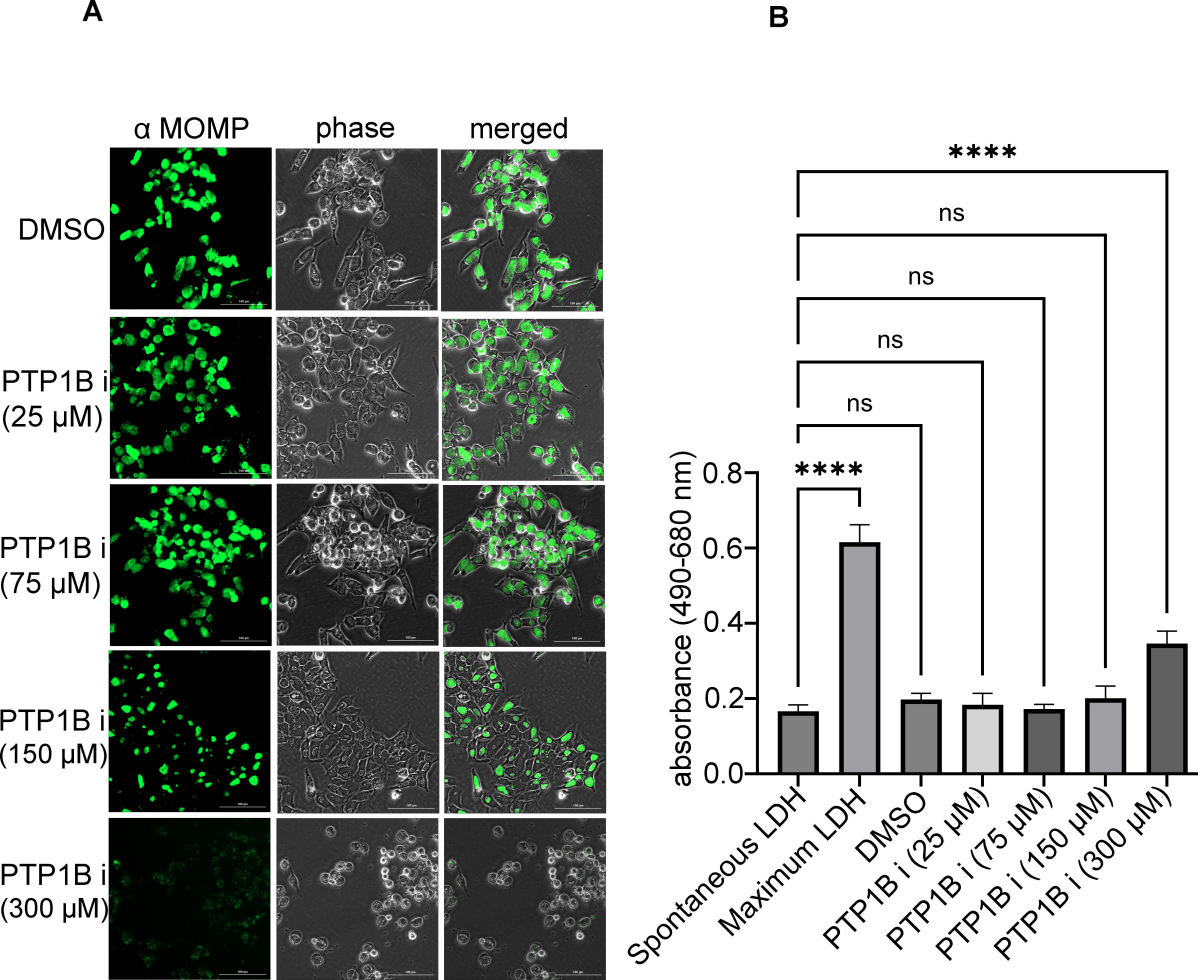
Effect of PTP1B inhibition on *Chlamydia trachomatis* inclusion formation and treatment-associated cytotoxicity. (**A**) HeLa cells were pre-treated with increasing concentrations of a PTP1B inhibitor and subsequently infected with *C. trachomatis* serovar L2 at a multiplicity of infection (MOI) of 5. At 36 h post-infection, cells were fixed with methanol and stained with a mouse monoclonal antibody against the chlamydial major outer membrane protein (MOMP), followed by goat anti-mouse secondary antibody conjugated to Alexa Fluor 488 (green) to visualize inclusions. Fluorescence and phase contrast images were acquired using the BioTek Cytation 5 imaging system. Scale bar, 100 µm. (**B**) To evaluate cytotoxicity associated with PTP1B inhibitor treatment, HeLa cells were treated with the PTP1B inhibitor for 27 h, and cytotoxicity was assessed using the Invitrogen CyQUANT LDH Cytotoxicity Assay. Absorbance was measured as an indicator of LDH release, with maximum and spontaneous release controls included. Statistical significance was determined using one-way ANOVA followed by Dunnett’s multiple comparisons test. ****P  <  0.0001; ns, not significant. Error bars represent standard deviation (SD).

The phosphoproteome of the infected cell changes during *C. trachomatis* entry and development, as both host and bacterial proteins are modified by host kinases (31, 32). Phosphoproteomics analysis of the chlamydial developmental cycle indicates that the phosphorylation of many cellular and bacterial targets is temporally controlled with phosphorylation levels both rising and falling (33). While much attention has previously been directed toward the role of kinases during chlamydial development, we sought to investigate the role of host cell phosphatases. To investigate the role of tyrosine phosphatase PTP1B in *C. trachomatis* development, HeLa cells were treated with increasing concentrations of a commercially available PTP1B chemical inhibitor, followed by infection with *C. trachomatis* elementary bodies (EBs) (Fig. 1A). *Chlamydia*-infected cells were fixed 40 h post-infection, and inclusions were visualized by immunofluorescence microscopy using an antibody specific for the chlamydial major outer membrane protein (MOMP). At the highest concentration tested (300  µM), visible signs of cytotoxicity, such as cell rounding and detachment, were observed. To ensure that the observed phenotypes were not due to cytotoxic effects of the inhibitor, we performed an LDH release assay following 27 h of treatment. Absorbance measurements indicated that only the highest concentration (300  µM) of the PTP1B inhibitor caused significant cytotoxicity, whereas 75  µM and 150  µM did not show any significant effect (Fig. 1B). These concentrations were therefore selected for downstream experiments.

**Fig 2:**
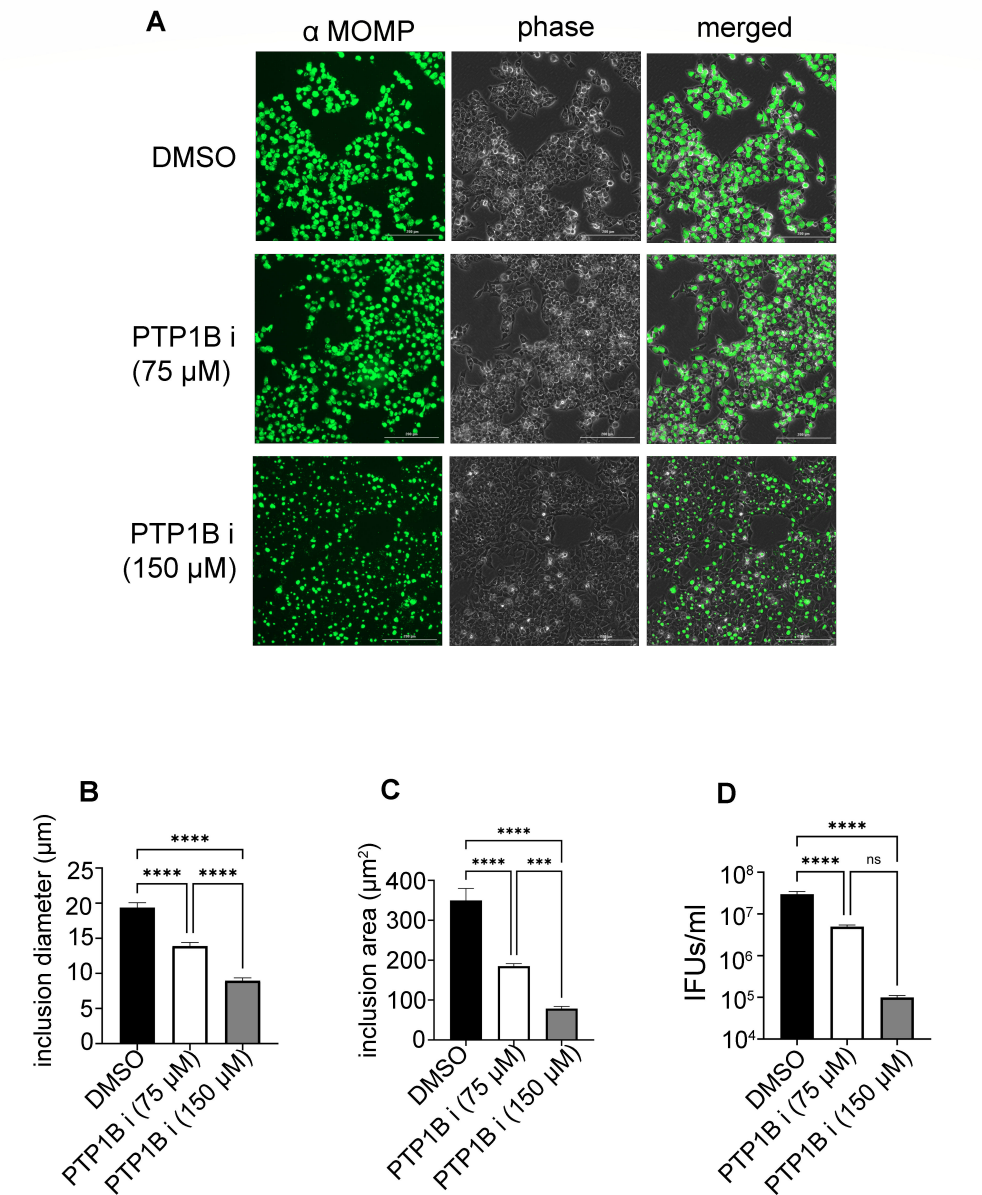
Chemical inhibition of PTP1B impairs *Chlamydia trachomatis* development. (**A**) HeLa cells treated with PTP1B chemical inhibitors (75 or 150 µM) or vehicle control (DMSO) were infected with *C. trachomatis* EBs. Forty hours post-infection, cells were fixed, and inclusions were visualized using mouse antibodies specific for the chlamydial major outer membrane protein (α MOMP) along with goat anti-mouse antibodies conjugated to Alexa 488 (green). Separate phase contrast (phase) and merged images are provided for each field of view. One representative image (out of 15–20 per condition) is provided for each treatment. Scale bar, 200 µm. (**B–C**) Quantification of the mean inclusion diameter (µm) and mean inclusion area (µm²) across treatment conditions. Error bars represent SD. (**D**) Quantification of the chlamydial development cycle by measuring the production of infectious progeny (inclusion-forming units [IFUs/mL]) harvested after 36–42 h of treatment. The number of viable EBs per mL was determined by infecting HeLa cells with serially diluted lysates and quantifying the formation of inclusions after 24 h. Data represent the mean of the three independent experiments. Error bars represent SD. Statistical significance was determined using one-way ANOVA followed by Tukey’s multiple comparisons test. ***P < 0.001; ****P < 0.0001; ns, not significant.

Compared to the DMSO control, PTP1B inhibition led to a dose-dependent reduction in *C. trachomatis* inclusion size, with treated cells displaying smaller and less defined inclusions (Fig. 2A). Quantification of captured immunofluorescence images confirmed a significant decrease in both mean inclusion diameter and area in PTP1B-inhibitor treated cells (Fig. 2B and C).

To determine whether impaired inclusion development observed for the PTP1B inhibitor-treated cells affected EB production, infectious progeny were quantified by infecting HeLa cells with EBs harvested 36–42 h post-treatment with and without PTP1B inhibitor. Cells treated with the PTP1B inhibitor exhibited significantly reduced inclusion-forming units (IFUs/mL) relative to the control, indicating diminished bacterial replication (Fig. 2D). These findings demonstrate that chemical inhibition of PTP1B disrupts *C. trachomatis* inclusion development, underscoring a potential regulatory role for PTP1B in host-pathogen interactions.

### PTP1B inhibition does not affect *Chlamydia trachomatis* invasion

**Fig 3:**
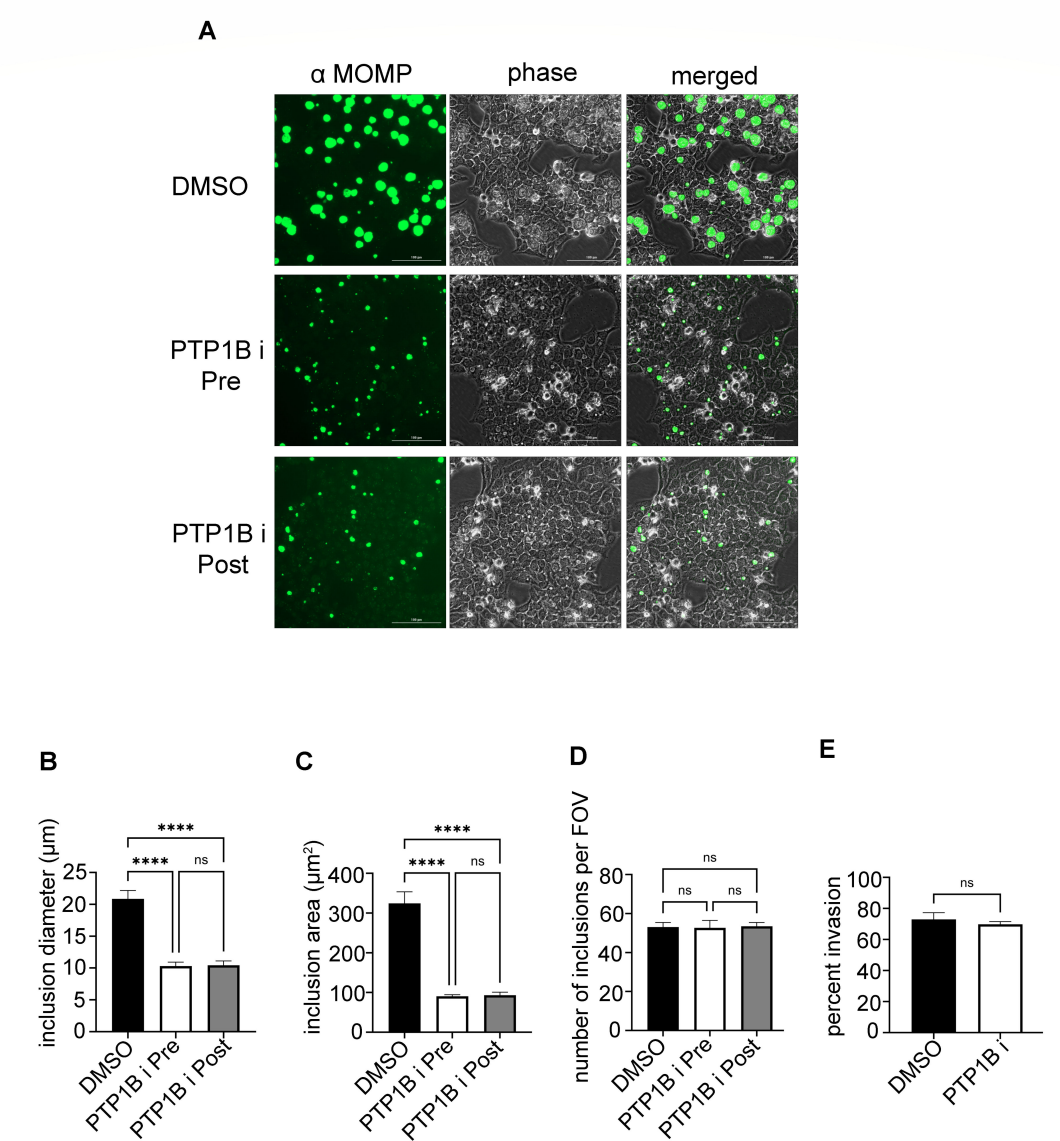
PTP1B inhibition does not affect *Chlamydia trachomatis* invasion. (**A**) HeLa cells were treated with 150 µM PTP1B inhibitor (PTP1Bi) either 2 h before *C. trachomatis* infection (Pre), 2 h after infection (Post), or with DMSO alone before infection (DMSO). At 40 h post-infection, cells were fixed, and inclusions were visualized using mouse antibodies specific for the chlamydial major outer membrane protein (α MOMP) along with goat anti-mouse antibodies conjugated to Alexa 488 (green). Separate phase contrast (phase) and merged images are provided for each field of view. One representative image is shown for each treatment. Scale bar, 100 µm. (**B–D**) Quantification of *C. trachomatis* inclusion size and number across conditions. Inclusion diameter (**B**), inclusion area (**C**), and the number of inclusions per field of view (**D**) were measured. Error bars represent SD. (**E**) Invasion assay. HeLa cells were pre-treated with DMSO or PTP1B inhibitor (150 µM) for 2 h and then infected with red fluorescent *C. trachomatis* EBs (pre-labelled with Cell Tracker dye). At 90 min post-infection, cells were fixed with 4% paraformaldehyde for 15 min and stained for extracellular EBs using a mouse anti-*Chlamydia* LPS antibody followed by Alexa 488-conjugated goat anti-mouse secondary antibody. The percentage of invaded bacteria was calculated by comparing total (red) versus extracellular (green) bacteria. At least 15 fields of view were analyzed per condition. Error bars represent SD. Data represent mean of the three independent experiments. Statistical significance was determined using one-way ANOVA with Tukey’s multiple comparisons test for (**B–D**) and an unpaired *t*-test for (**E**) ****P < 0.0001; ns, not significant.

Given our findings that PTP1B inhibition reduces *Chlamydia trachomatis* inclusion size and progeny, we sought to determine whether this effect was due to impaired bacterial invasion or defects in intracellular development post-entry. To accomplish this, we compared *C. trachomatis* inclusion numbers and development in HeLa cells treated with the PTP1B inhibitor either 2 h before (*pre-treatment*) or 2 h after (*post-treatment*) infection with *C. trachomatis*. Cells were fixed at 40 h post-infection, and inclusions were visualized by staining with MOMP-specific antibodies (Fig. 3A).

Quantification of inclusion size revealed a significant reduction in inclusion diameter (Fig. 3B) and inclusion area (Fig. 3C) in both the pre-treatment and post-treatment groups compared to the DMSO controls. However, no significant difference in size was observed between the pre-treatment and post-treatment groups, indicating that PTP1B inhibition impairs inclusion growth regardless of whether it occurs before or after bacterial entry. In contrast, the number of inclusions per field of view remained unchanged across all conditions (Fig. 3D), suggesting that PTP1B inhibition does not affect *C. trachomatis* invasion. Additionally, an invasion assay using CMTPX-red-labeled EBs confirmed no significant difference in bacterial entry between PTP1B-inhibited and control cells (Fig. 3E). Together, these findings suggest that PTP1B activity is important for the intracellular development of *C. trachomatis* but is dispensable for bacterial entry into host cells.

### PTP1B knockdown impairs *Chlamydia trachomatis* intracellular development and reduces infectivity

**Fig 4:**
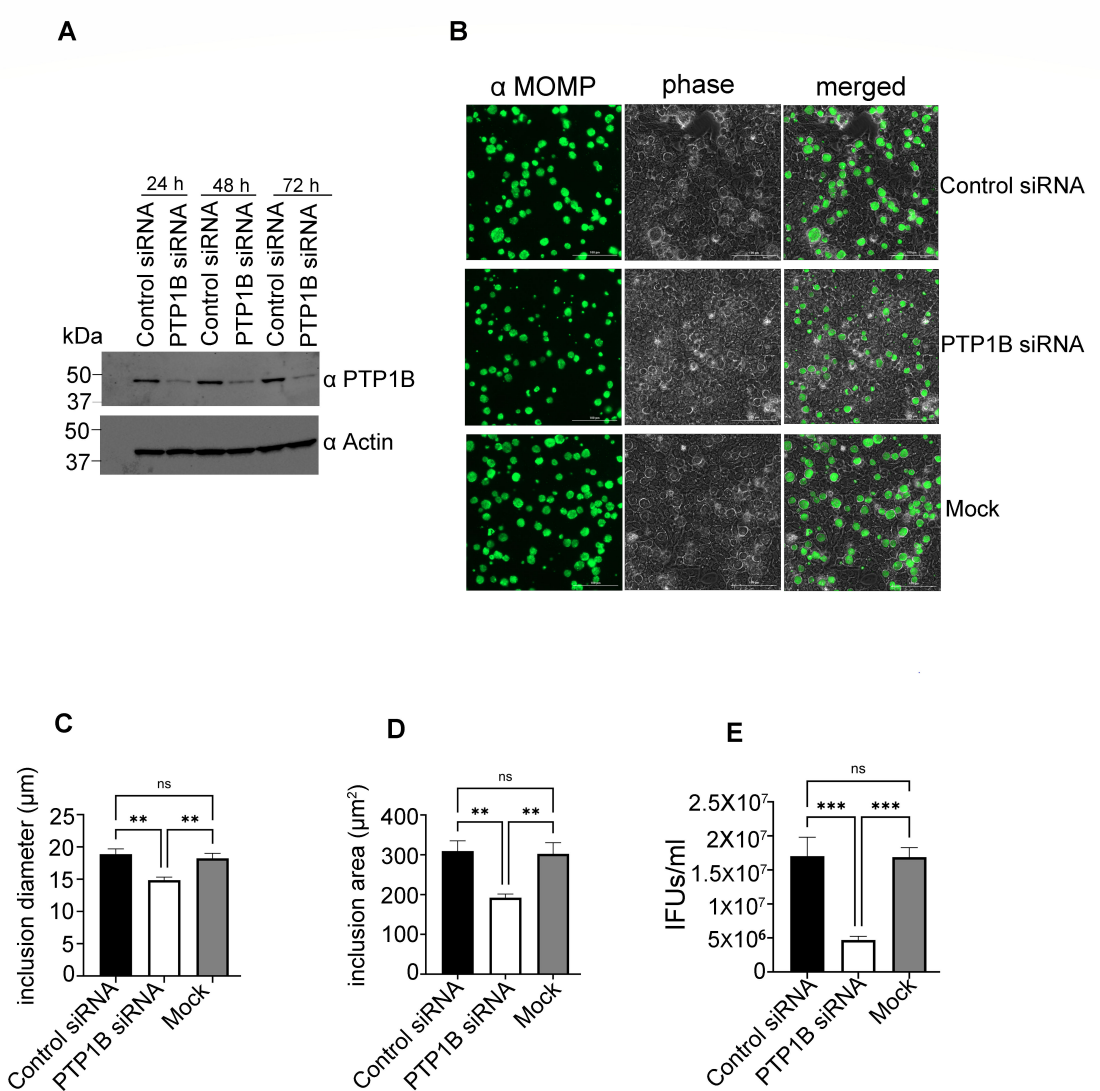
PTP1B knockdown impairs *C. trachomatis* inclusion development and reduces infectivity. (**A**) HeLa cells were transfected with PTP1B-targeting siRNA or control siRNA, and lysates were collected at 24, 48, and 72 h post-transfection. Samples were resolved by SDS-PAGE and subjected to immunoblotting with antibodies specific for PTP1B (α PTP1B) and actin (α Actin) as a loading control. (**B**) Forty hours post-infection, cells were fixed, and inclusions were visualized using mouse antibodies specific for the chlamydial major outer membrane protein (α MOMP) and goat anti-mouse antibodies conjugated to Alexa 488 (green). Separate phase contrast (phase) and merged images are provided for each condition. One representative image is shown. Scale bar, 100 µm. (**C–E**) Quantification of the mean inclusion diameter (µm) (**C**), mean inclusion area (µm^2^) (**D**), and bacterial infectivity based on inclusion-forming units (IFUs/mL) (**E**). Infectious progeny were harvested at 40 h post-infection, and infectivity was determined by reinfecting fresh HeLa cells with serially diluted lysates and quantifying inclusion formation. Data represent the mean of the three independent experiments. Error bars represent SD. Statistical significance was determined using one-way ANOVA followed by Tukey’s multiple comparisons test. ***P < 0.001; **P  <  0.01; ns, not significant.

To extend our chemical inhibitor findings and to directly assess the role of PTP1B, we utilized an siRNA-based approach to deplete endogenous PTP1B in HeLa cells. Western blot analysis confirmed efficient and stable depletion of PTP1B protein levels at 24, 48, and 72 h post-transfection with PTP1B-targeting siRNAs, while control siRNA-treated cells retained stable PTP1B expression (Fig. 4A). As PTP1B protein levels were reduced as early as 24 h post-transfection, cells were infected with *C. trachomatis* 24 h after knockdown and fixed at 40 h post-infection, and inclusions were visualized by immunofluorescence microscopy. PTP1B-depleted cells exhibited smaller inclusions compared to control siRNA and mock-treated cells (Fig. 4B). Quantitative image analysis confirmed a significant reduction in both mean inclusion area and diameter in the knockdown group (Fig. 4C and D). To assess whether the reduction in inclusion size interfered with EB production, we quantified infectious progeny by measuring inclusion-forming units (IFUs/mL). Consistent with reduced inclusion size, PTP1B knockdown resulted in significantly lower IFUs compared to the control groups (Fig. 4E). Together, these findings demonstrate that specific depletion of endogenous PTP1B disrupts *C. trachomatis* intracellular development and replication, reinforcing the role of PTP1B as a host factor that promotes chlamydial growth.

### PTP1B overexpression promotes *Chlamydia trachomatis* inclusion development and increases bacterial yield

**Fig 5:**
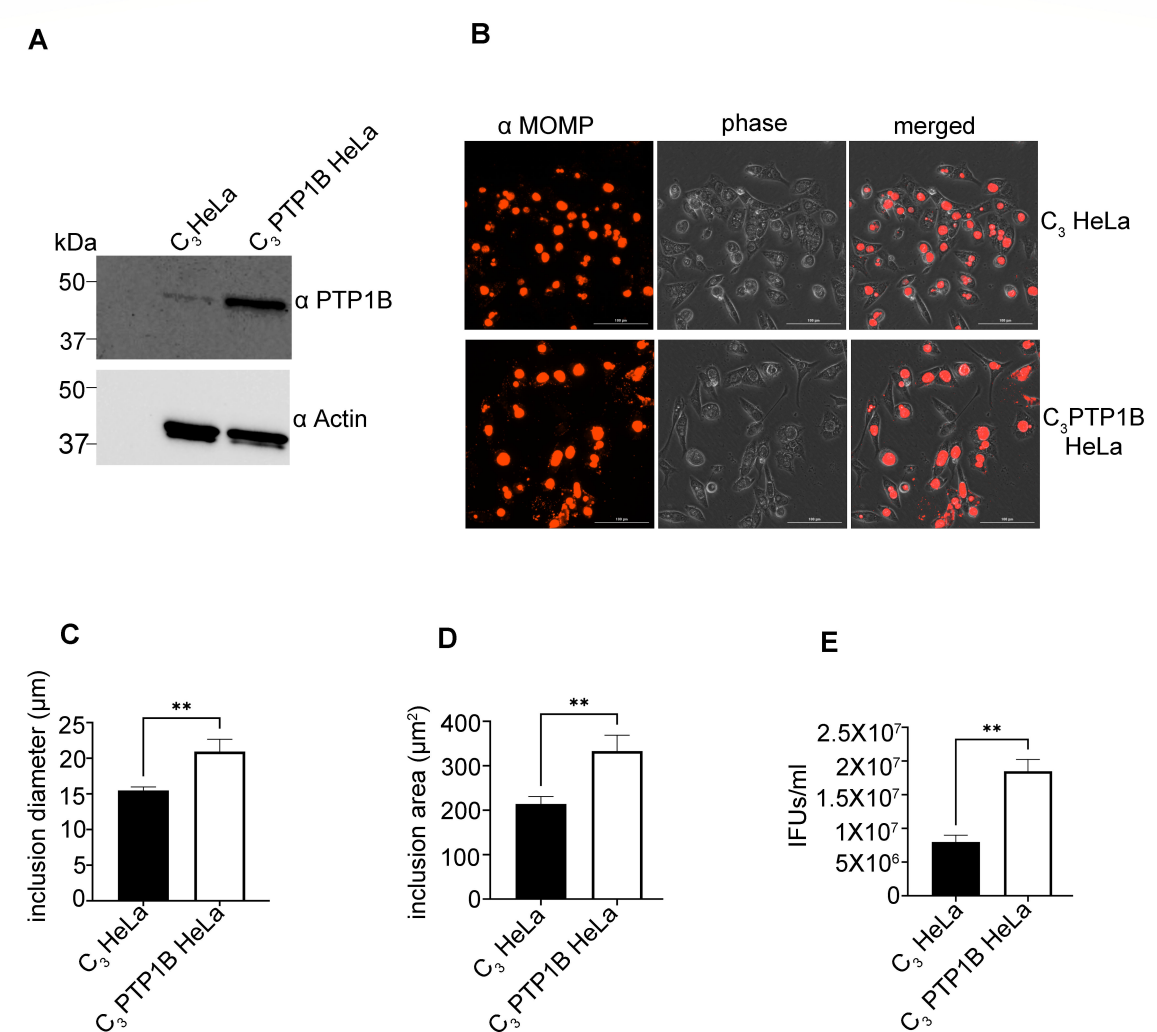
PTP1B overexpression enhances *Chlamydia trachomatis* development and infectivity. (**A**) HeLa cells were transfected with either an empty pEGFPC₃ vector (C₃ HeLa) or a PTP1B-expressing construct (C₃ PTP1B HeLa). Forty-eight hours post-transfection, whole-cell lysates were collected and subjected to SDS-PAGE followed by immunoblotting with antibodies specific for PTP1B (α PTP1B) and actin (α Actin) as a loading control. (**B**) Forty hours post-infection with *C. trachomatis*, cells were fixed and stained using mouse antibodies specific for the chlamydial major outer membrane protein (α MOMP) and goat anti-mouse antibodies conjugated to Alexa 594 (red). Separate phase contrast (phase) and merged images are shown. One representative field of view is presented for each condition. Scale bar, 100 µm. (**C–E**) Quantification of the mean inclusion diameter (µm) (**C**), mean inclusion area (µm²) (**D**), and bacterial infectivity based on inclusion-forming units (IFUs/mL) (**E**). Infectious progeny were harvested at 40 h post-infection, and infectivity was determined by reinfecting fresh HeLa cells with serially diluted lysates and quantifying inclusion formation. Data represent the mean of the three independent experiments. Error bars represent SD. Statistical significance was determined using unpaired *t*-tests. **P < 0.01.

To assess whether elevated PTP1B levels impact *C. trachomatis* intracellular development, we cloned the coding sequence of human PTP1B into the mammalian expression vector pEGFPC₃ and transfected HeLa cells. We selected stable cells overexpressing PTP1B with G418 (Geneticin). Stable overexpression of PTP1B in transfected cells (C₃ PTP1B HeLa) was confirmed by immunoblot, which demonstrated increased PTP1B protein levels compared to vector-only control cells (C₃ HeLa) (Fig. 5A). Transfected cells were infected with *C. trachomatis*, fixed 40 h post-infection, stained with antibodies specific for the chlamydial major outer membrane protein (MOMP), and visualized by fluorescence microscopy. Compared to control cells, PTP1B-overexpressing cells exhibited larger and more prominent inclusions (Fig. 5B). Quantitative analysis revealed a significant increase in both mean *C. trachomatis* inclusion diameter and area in C₃ PTP1B-transfected cells compared to control-transfected cells (Fig. 5C and D). To evaluate whether PTP1B overexpression enhances bacterial replication, infectious progeny were quantified by measuring inclusion-forming units (IFUs/mL). C₃ PTP1B-transfected cells produced significantly more infectious EBs than vector control-transfected cells (Fig. 5E). Albeit a small but statistically significant increase, these data indicate that elevated levels of PTP1B promote intracellular growth and replication of *C. trachomatis*, further supporting its positive regulatory role during infection.

### Recombinant PTP1B dephosphorylates the *Chlamydia trachomatis* early effector Tarp

**Fig 6:**
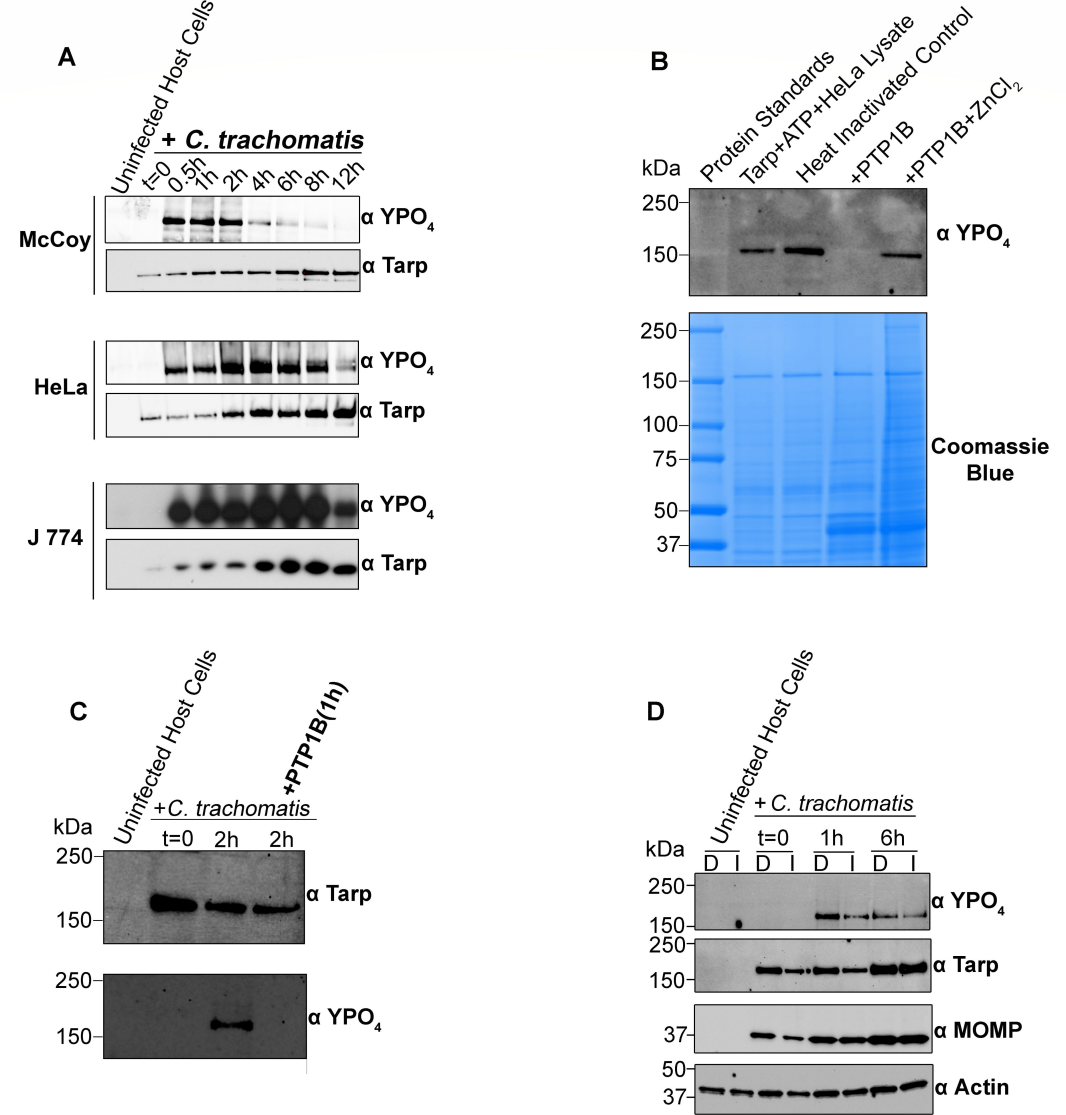
Tarp phosphorylation kinetics and dephosphorylation by recombinant PTP1B. (**A**) Time course analysis of Tarp phosphorylation during *C. trachomatis* infection in different cell lines. McCoy, HeLa, and J774 cells were infected with *C. trachomatis* L2 (MOI ~ 50–100) and harvested at the indicated time points post-infection. Lysates were resolved by SDS-PAGE and immunoblotted with phosphotyrosine-specific (α YPO₄) and Tarp-specific (α Tarp) antibodies to assess phosphorylation dynamics. Uninfected host cell lysates served as controls. (**B**) Dephosphorylation of recombinant Tarp by recombinant PTP1B. Purified recombinant Tarp was phosphorylated in the presence of ATP and HeLa lysate and then heat-inactivated to stop kinase activity. Recombinant PTP1B was added for 1 h in the presence or absence of ZnCl₂, and samples were resolved by SDS-PAGE, stained with Coomassie blue, and immunoblotted with phosphotyrosine-specific (αYPO₄) antibodies. (**C**) Dephosphorylation of native Tarp by recombinant PTP1B. Extracts from *C. trachomatis*-infected HeLa cells (2 h post-infection) were incubated with recombinant PTP1B for 1 h. Extracts from uninfected HeLa cells served as controls. Proteins were resolved by SDS-PAGE and immunoblotted with phosphotyrosine (α YPO_4_) and Tarp-specific (α Tarp) antibodies. Molecular weight markers are shown in kilodaltons (kDa). (**D**) Tarp phosphorylation kinetics during *C. trachomatis* infection of HeLa cells treated with either DMSO control (D) or PTP1B inhibitor (I). Cells were harvested at 0, 1, and 6 h post-infection. Lysates were analyzed by SDS-PAGE and immunoblotting with anti-phosphotyrosine (α YPO₄), anti-Tarp (α Tarp), anti-MOMP (α MOMP), and anti-actin (α Actin) antibodies. Actin served as a loading control.

Early chlamydial effectors such as Tarp are phosphorylated by host tyrosine kinases soon after translocation into a mammalian host cell during EB entry (20). To investigate the phosphorylation kinetics of Tarp during *Chlamydia trachomatis* infection, we examined Tarp phosphorylation levels at discrete time points between 0 and 12 h post infection in McCoy, HeLa, and J774 macrophage cell lines. As seen previously, immunoblot analysis demonstrated that Tarp is rapidly phosphorylated upon infection, with phospho-Tarp levels peaking early and declining over time depending on the tissue culture cell type employed (Fig. 6A). Interestingly, total Tarp protein levels increased up to 4 h post-infection and then stabilized, indicating that the observed changes in phospho-Tarp levels were due to dynamic phosphorylation rather than new Tarp synthesis. *De novo* Tarp expression occurs late in the developmental cycle, and therefore, Tarp levels observed early in the infection are EB-derived. The observation that the Tarp effector undergoes active dephosphorylation prompted us to examine whether PTP1B contributes to the regulation of phospho-Tarp levels.

To investigate whether PTP1B is capable of targeting chlamydial effector proteins, we tested whether recombinant PTP1B is able to dephosphorylate Tarp, a key early effector that undergoes tyrosine phosphorylation during *Chlamydia trachomatis* entry. First, recombinant PTP1B was expressed in *E. coli* and purified by affinity chromatography. Coomassie-stained SDS-PAGE and immunoblot confirmed the successful purification of soluble recombinant PTP1B protein (~45 kDa) (Fig. S1A). To assess phosphatase activity, purified recombinant Tarp was phosphorylated *in vitro* using HeLa cell lysate and ATP. Following heat inactivation of the lysate to eliminate residual kinase activity, recombinant PTP1B was added to the reaction (1 h) with or without ZnCl_2_. ZnCl_2_ was used as an additional negative control for PTP1B activity due to the ability of Zinc ions to inhibit the activity of PTP1B (34). Immunoblot analysis using phosphotyrosine-specific antibodies revealed an absence of detectable phospho-Tarp following treatment with recombinant PTP1B (Fig. 6B), indicating dephosphorylation of Tarp. In contrast, phospho-Tarp was readily detectable in the sample treated with PTP1B in the presence of zinc chloride, suggesting that zinc inhibited PTP1B activity. We next examined the ability of PTP1B to dephosphorylate native Tarp. Lysates from HeLa cells infected with *C. trachomatis* for 2 h were incubated with recombinant PTP1B for 1 h. Immunoblot revealed an absence of detectable phospho-Tarp in PTP1B-treated samples, while total Tarp levels remained unchanged (Fig. 6C).

To further explore the role of PTP1B during infection, we examined whether PTP1B inhibition altered native Tarp phosphorylation dynamics. HeLa cells were treated with DMSO or PTP1B inhibitor (150 µM) and infected with *C. trachomatis*, and cell lysates were collected at 0, 1, and 6 h post-infection. Immunoblot showed that phospho-Tarp levels followed a parallel trajectory in both groups, suggesting that inhibition of PTP1B did not substantially affect Tarp phosphorylation kinetics (Fig. 6D). However, total Tarp levels were transiently decreased at the early infection stage in inhibitor-treated cells and returned to baseline by 6 h. This transient decrease was likely attributable to the chemical stimulation of *Chlamydia trachomatis* elementary bodies (EBs) by the PTP1B inhibitor, which we found can induce the release of Tarp into the extracellular milieu even in the absence of host cell contact (Fig. S1B). Nevertheless, this does not alter the conclusion that PTP1B inhibition did not significantly impact the phosphorylation kinetics of Tarp during infection. Together, our findings demonstrate that recombinant PTP1B can directly dephosphorylate Tarp and suggest that host phosphatases have the capacity to modulate chlamydial effector function, potentially influencing early host-pathogen interactions.

## DISCUSSION

The *Chlamydia trachomatis* developmental cycle requires microbial control of the infected host cell to subvert host defenses and to promote nutrient acquisition (35). Such control is accomplished in part by a collection of bacterial effectors whose purpose is to commandeer host signaling and metabolic pathways (36–38). While the contribution of host kinases to *C. trachomatis* pathogenesis has been extensively studied—including their roles in cytoskeletal remodeling, inclusion trafficking, and effector phosphorylation (18, 39)—the role of host phosphatases remains largely uncharacterized. Given the extensive rewiring of host and bacterial phosphorylation networks during infection, understanding how host phosphatases contribute to chlamydial development represents an important and underexplored question. Interestingly, the importance of bacterial phosphatase activity in *C. trachomatis* development has previously been demonstrated. Claywell et al. identified a chlamydial phosphatase, CppA, whose activity contributed to inclusion stability and development (23). However, the potential regulatory roles of host phosphatases during infection remain unknown.

 In this study, we identify the host protein tyrosine phosphatase PTP1B as a positive regulator of chlamydial intracellular development. Using complementary approaches—chemical inhibition, siRNA-mediated knockdown, and overexpression—we demonstrated that PTP1B plays a significant role in promoting *C. trachomatis* intracellular development. Treatment of host cells with a specific PTP1B inhibitor resulted in a marked reduction in the size of chlamydial inclusions as well as a substantial decrease in the yield of infectious elementary bodies. These inhibitory effects were observed whether cells were treated with the inhibitor prior to infection or after bacterial entry, suggesting that PTP1B functions primarily during the intracellular stages of the *Chlamydia* developmental cycle. To determine whether PTP1B also influences bacterial entry, we performed invasion assays under inhibitor-treated conditions. These experiments revealed no measurable effect on bacterial internalization, indicating that PTP1B activity is dispensable for the entry process itself. Silencing of PTP1B by siRNA yielded similar phenotypes, reinforcing the conclusion that PTP1B is required for efficient bacterial replication within host cells. In contrast, ectopic overexpression of wild-type PTP1B enhanced both inclusion expansion and EB production, further validating its role as a host factor that supports chlamydial growth.

To investigate the mechanism by which PTP1B supports chlamydial growth, we examined its interaction with the chlamydial effector Tarp, which is phosphorylated by host tyrosine kinases and contributes to actin remodeling during bacterial entry (19, 20, 40). *In vitro* assays demonstrated that PTP1B can dephosphorylate both recombinant and native forms of phosphorylated Tarp, suggesting that Tarp is a possible substrate of PTP1B. However, inhibition of PTP1B in infected cells did not significantly alter Tarp phosphorylation kinetics, suggesting that PTP1B is not the primary regulator of Tarp phosphorylation during infection. This may be due to functional redundancy among host phosphatases or spatial compartmentalization of PTP1B. A transient reduction in total Tarp levels was observed early during infection in PTP1B-inhibited cells. This observation was likely due to altered effector secretion during the initial host-pathogen interaction. Cell-free elementary bodies exposed to the PTP1B inhibitor released detectable amounts of Tarp. However, this effect did not persist post-infection or affect downstream phosphorylation, suggesting that it may reflect a minor perturbation in effector release rather than a major regulatory role. Nonetheless, the observation raises the possibility that PTP1B may influence the early dynamics of effector delivery or stabilization in a context-dependent manner.

 Together, these results establish PTP1B as a host factor that supports *C. trachomatis* intracellular replication. Although prior genome-wide siRNA screens, such as that by Rother et al. (41), included numerous phosphatases—including PTP1B (PTPN1), PPP2CA, PPP1CA, PTPN2, and PTPRJ—none were highlighted as key hits in the published analysis, leaving their potential roles in *Chlamydia* infection largely unexplored. Our findings suggest that host phosphatases—such as PTP1B—may represent an underappreciated layer of regulation, possibly acting in parallel or compensatory signaling pathways. Our study is the first to functionally validate its role during *C. trachomatis* infection. Although our biochemical assays demonstrated that PTP1B is capable of dephosphorylating both recombinant and native Tarp, inhibition of PTP1B during infection did not noticeably alter Tarp phosphorylation dynamics. This suggests that Tarp is unlikely to be a major physiological substrate of PTP1B in the context of infection. Nonetheless, these findings highlight PTP1B’s ability to engage with chlamydial effectors, underscoring its potential to influence bacterial–host interactions through other, yet unidentified, targets.

In light of our findings, it is worth considering whether other host phosphatases may similarly contribute to *C. trachomatis* development. While PTP1B is a member of the non-receptor type protein tyrosine phosphatase (PTPN) family, several other tyrosine phosphatases are expressed in epithelial cells and could potentially influence infection dynamics (42–44). Moreover, serine/threonine phosphatases—though not expected to directly regulate tyrosine-phosphorylated effectors like Tarp—may modulate other bacterial or host targets critical to infection. For example, chlamydial proteins have been shown to undergo diverse post-translational modifications, including serine/threonine phosphorylation (17, 18), which may be influenced by these phosphatases. Future studies aimed at systematically evaluating both tyrosine and serine/threonine phosphatases may reveal broader host phosphatase networks that modulate chlamydial pathogenesis.

Beyond its mechanistic role, PTP1B is a clinically relevant target. Several PTP1B inhibitors have been developed for non-infectious diseases and have reached human clinical trials. Ertiprotafib, developed by Wyeth, was the first PTP1B inhibitor to be tested for treatment of type 2 diabetes and obesity (45). Other inhibitors, including KQ-791, trodusquemine (MSI-1436), and ABBV-CLS-484, are currently being evaluated for efficacy in trials for metabolic disorders and cancer (46, 47). Over eight PTP1B-targeting compounds have entered clinical development (48). Preclinical studies also suggest neuroprotective benefits from PTP1B inhibition, including protection from age-related neurodegeneration (49–51). The existence of this drug pipeline opens the possibility of repurposing PTP1B inhibitors as host-directed therapeutics against intracellular pathogens like *C. trachomatis*. While further validation is needed, our findings support this concept as a potential avenue for combatting persistent or antibiotic-refractory infections.

These findings presented here contribute to a broader recognition that host phosphatases—long overshadowed by kinases in infection biology—play underappreciated but critical roles in host-pathogen interactions. Our study highlights the value of investigating phosphatase signaling networks in the context of infection. Future research will aim to identify additional PTP1B substrates during *C. trachomatis* infection and clarify how phosphatase activity is temporally and spatially regulated within infected cells. Such work may reveal novel regulatory circuits that are exploitable for therapeutic intervention and expand our understanding of host-directed strategies to restrict intracellular bacterial growth.

## MATERIALS AND METHODS

### Organisms and cell culture

*Chlamydia trachomatis* serovar L2 (LGV 434/Bu) was propagated in HeLa 229 and McCoy cells and purified by density gradient centrifugation using diatrizoate meglumine and diatrizoate sodium as previously described (52). HeLa, McCoy, and J774 cell lines were obtained from the American Type Culture Collection (ATCC) and maintained in Dulbecco’s Modified Eagle Medium (DMEM; Gibco) supplemented with 10% fetal bovine serum (FBS; Gibco) and 1% L-glutamine (Gibco). All cell lines were cultured at 37°C in a humidified incubator with 5% CO₂. HeLa cells were used for the majority of experiments unless otherwise noted.

### PTP1B inhibitor treatment

PTP1B inhibition was performed using a selective small-molecule inhibitor (CAS-765317-72-4, Millipore Sigma). An initial dose–response titration was conducted by treating HeLa cells with inhibitor concentrations ranging from 25 to 300 µM for 2 h prior to infection, in order to assess effects on host cell morphology and *Chlamydia trachomatis* inclusion development.

For pre-treatment protocols, cells were incubated with the inhibitor for 2 h prior to infection, and the inhibitor-containing medium was maintained throughout the 40-hour infection period unless otherwise specified.

In post-infection treatment experiments, HeLa cells were infected with *C. trachomatis* serovar L2 for 2 h, after which the inoculum was removed and replaced with fresh medium containing either DMSO (vehicle control at the same concentration as inhibitor) or PTP1B inhibitor at the indicated concentrations. Cells were then incubated for the remainder of the 40-hour infection period prior to fixation and analysis.

### LDH cytotoxicity assay

LDH release was quantified using the Invitrogen CyQUANT LDH Cytotoxicity Assay Kit according to the manufacturer’s instructions. Briefly, HeLa cells were seeded in 96-well plates (3,000 cells per well) 24 h prior to treatment and incubated with the PTP1B inhibitor (25 µM–300 µM) for 27  h. For controls, maximum LDH release wells were treated with 10× lysis buffer, and spontaneous LDH release wells received sterile water for 45  min before supernatant collection.

At the end of the treatment period, culture supernatants were collected from each well and transferred to fresh assay wells. LDH reaction mixture was added as directed by the kit protocol, incubated per the manufacturer’s instructions at room temperature (protected from light), and the reaction was stopped with the provided stop solution. Absorbance was recorded at 490  nm with 680  nm as the reference wavelength. Absorbance was analyzed as an indicator of LDH release. For each well, background-corrected absorbance was calculated as *A*490–*A*680. For data processing, technical replicates were averaged, and treatment groups were compared to vehicle (DMSO) controls.

### *Chlamydia trachomatis* infection and indirect immunofluorescence

HeLa cells were seeded in 6-well plates at a density of 2 × 10^5^ cells per well and pre-treated the following day with PTP1B inhibitor (75–150 µM) or DMSO for 2 h. After pre-treatment, cells were infected with *Chlamydia trachomatis* serovar L2 at a multiplicity of infection (MOI) ranging from 0.1 to 5 and incubated at 37°C with 5% CO_2_ for 40 h. In case of siRNA treatment or PTP1B transfections, cells were infected after a suitable treatment duration (24–72 h).

 At 36–42 h post-infection, cells were fixed with methanol for 15 min at room temperature and washed three times with 1× phosphate-buffered saline (PBS). Fixed cells were blocked with 10% bovine calf serum (Gibco) for 1 h and incubated with a mouse monoclonal antibody against *C. trachomatis* major outer membrane protein (MOMP) (Invitrogen) at 1:500 dilution for 1 h at room temperature, followed by three to four PBS washes and incubation with an Alexa Fluor–conjugated goat anti-mouse IgG secondary antibodies Alexa 488 or Alexa 594 (Invitrogen) at 1:500 dilution for 1 h in the dark. Fluorescence images were acquired using the Biotek Cytation 5 imaging system using 40× magnification, and inclusion diameters and area were measured using the Gen5 software. Ten to fifteen fields of view were analyzed per condition.

### Inclusion forming unit assay

HeLa cells seeded at 2 × 10^5^ cells per well were infected with *C. trachomatis* serovar L2 at a multiplicity of infection (MOI) ranging from 0.1 to 5 and incubated at 37°C for 36–42 h. Cells were scraped, and the entire contents of each well (cells plus media) were collected. Samples were subjected to brief sonication to release elementary bodies. The resulting lysates were serially diluted in Hanks’ Balanced Salt Solution (HBSS) and used to infect fresh monolayers of HeLa cells seeded in 24-well plates. After a 24-hour secondary infection period, cells were fixed with methanol for 15 min, washed with PBS, blocked with 10% bovine calf serum, and stained using a mouse monoclonal antibody against *C. trachomatis* MOMP (1:500 dilution), followed by an Alexa Fluor 488–conjugated goat anti-mouse secondary antibody (1:500 dilution). Fluorescence images were acquired using the Biotek Cytation 5 imaging system, and inclusion-forming units (IFUs) were quantified using the Gen5 software by analyzing 15 random fields per well at 20× magnification.

### *C. trachomatis* invasion assay

*Chlamydia trachomatis* elementary bodies (EBs) were labeled with CellTracker Red CMTPX Dye (Invitrogen) during propagation in HeLa cells, as previously described (53). For invasion assays, CMTPX-labeled EBs were harvested and used to infect HeLa cells seeded in 24-well tissue culture plates. Cells were grown in Dulbecco’s Modified Eagle Medium (DMEM) supplemented with 10% fetal bovine serum (FBS) and 1% L-glutamine for 24 h prior to infection. To synchronize the infections, plates were pre-chilled on ice for 5 min. CMTPX-labeled EBs were added to the wells at a multiplicity of infection (MOI) of ~10 and allowed to attach for 20 min while the plates remained on ice. After attachment, pre-warmed DMEM containing 10% FBS was added, and plates were transferred to 37°C for 90 min to allow internalization. Following incubation, media was removed, and cells were fixed with 4% paraformaldehyde (PFA) for 15 min at room temperature. Cells were not permeabilized. To label extracellular EBs, a mouse monoclonal anti-*Chlamydia* LPS antibody (Invitrogen) was used at a 1:100 dilution for 1 h at room temperature. After four PBS washes, Alexa Fluor 488-conjugated anti-mouse secondary antibody was applied (dilution 1:500) for 1 h. After final washes, fluorescence imaging was performed directly in the 24-well plates using the Biotek Cytation 5 imaging system. At least 15 fields of view per well were acquired under identical exposure settings. Quantification of invasion efficiency was conducted using Gen5 software (Agilent). Red fluorescence (CMTPX) marked total EBs, while green fluorescence indicated extracellular EBs. The percentage of internalized EBs was calculated using the formula: [((red − green)/red) × 100] = % Invasion.

### siRNA-mediated knockdown of PTP1B

PTP1B knockdown was performed using small interfering RNAs (siRNAs) synthesized by Integrated DNA Technologies (IDT). Cells were seeded in antibiotic-free DMEM supplemented with 10% FBS and allowed to reach 50–60% confluency prior to transfection. Transfections were carried out using Lipofectamine 3000 (Thermo Scientific) according to the manufacturer’s protocol. Briefly, cells were transfected with 20–60 nM siRNAs targeting human *PTPN1* or a non-targeting negative control siRNA (Table S1). To assess knockdown efficiency, cells were lysed directly in 5× SDS sample buffer at 24, 48, and 72 h post-transfection.

### Cloning and protein expression

The in-frame amino-terminal glutathione-S-transferase (GST) fusion protein for PTP1B was generated by PCR amplifying the PTP1B gene from cDNA from human dermal fibroblast cells (ATCC). PCR was performed with synthesized oligonucleotide primers (Integrated DNA Technologies) engineered with EcoRI and NotI linkers ( Table S1). PCR products were purified, digested with restriction enzymes (New England BioLabs), and subcloned into linearized pGEX-6p-1 to generate translational fusions with GST. The PTP1B gene was also cloned into mammalian expression vector pEGFP-C3 (Clontech) to allow for ectopic expression of PTP1B in HeLa cells. PCR was performed with DNA primers engineered with NheI and BamHI linkers (Table S1). Protein expression and purification were performed according to the procedures outlined for glutathione Sepharose 4B in the Bulk GST Purification Module (Cytiva). In some experiments, the GST tag was removed prior to kinase and phosphatase assays by treatment with PreScission Protease according to the manufacturer’s recommendations (Cytiva).

### PTP1B overexpression by transient and stable transfection

HeLa cells were transfected (transient or stable) with PTP1B construct cloned into the pEGFPC_3_ vector (PTP1BC_3_) or with the empty pEGFPC_3_ vector (Clontech). Transfections were performed in 6-well plates (Costar) at ~50–60% confluency using Lipofectamine 3000 (Invitrogen), following the manufacturer’s protocol. For each well, 5  µg of plasmid DNA was mixed with Lipofectamine 3000 and P3000 reagent in Opti-MEM and added directly to the cells. For transient transfection, cells were incubated for 48 h post-transfection before being processed for downstream experiments. For stable transfection, G418 (geneticin) was added at 150 µg/mL 24 h post-transfection, and the selection medium was replaced every 24–36 h. Stably transfected cells were expanded and used for downstream applications once resistant populations were established.

### Tarp phosphorylation kinetics

To evaluate the temporal phosphorylation of the *Chlamydia trachomatis* effector protein Tarp during early infection, HeLa, McCoy, and J774 cells (2 × 10^5^ cells) were seeded in 6-well plates and infected with *C. trachomatis* serovar L2 at a multiplicity of infection (MOI) of 50–100. A synchronized infection was performed to ensure uniform bacterial entry: prior to infection, cells were placed on ice and infected with cold bacterial suspensions for 30 min, followed by the addition of warm DMEM to initiate synchronous internalization. Plates were then transferred to a 37°C incubator, and infections were allowed to proceed for the indicated durations. Cells were harvested at defined time points between 0 and 12 h post-infection (hpi) to capture dynamic changes in Tarp phosphorylation. At each time point, media was removed, and cells were lysed directly in 5× SDS sample buffer (62.5 mM Tris-HCl [pH 6.8], 10% glycerol, 2% SDS, 5% β-mercaptoethanol, 0.01% bromophenol blue). Lysates were boiled for 5 minutes and stored at −20°C until further analysis.

### SDS-PAGE and immunoblot analysis

Protein samples were prepared in 5× SDS sample buffer, heated at 95°C for 5 min, and resolved on 4–12% gradient polyacrylamide gels (Invitrogen). Following electrophoresis, gels were either stained with Imperial Protein Stain (Thermo Scientific) or used for protein transfer onto nitrocellulose membranes via a wet transfer system (Bio-Rad). Membranes were blocked in 5% non-fat dry milk prepared in 1× phosphate-buffered saline with 0.001% Tween-20 for 1 h at room temperature. Primary antibody incubations were performed overnight at 4°C in 1× PBST containing 5% non-fat dry milk. The following primary antibodies were used (dilution 1:1000): monoclonal anti-PTP1B (Santa Cruz Biotechnology), monoclonal anti-actin C4 (Millipore Sigma), monoclonal anti-pan actin clone **7A8.2.1** (Cytoskeleton), monoclonal anti-phosphotyrosine 4G10 (Millipore Sigma), and monoclonal *Chlamydia trachomatis* anti-MOMP (Invitrogen). Polyclonal rabbit antibodies against *Chlamydia trachomatis* L2 LGV 434 Tarp (CT456) were generated at Rocky Mountain Laboratories, as previously described (16). After three washes with 1× PBST, membranes were incubated with horseradish peroxidase (HRP)-conjugated goat anti-mouse or goat anti-rabbit secondary antibodies (Invitrogen) for 1 h at room temperature, followed by three to four additional washes with 1× PBST. Blots were developed using Protein Biology chemiluminescent substrate (Thermo Scientific, Pierce), and signals were visualized using a Bio-Rad imaging system or by exposure to autoradiographic film.

### Recombinant Tarp dephosphorylation assay

Recombinant N-terminal domain of Tarp was incubated with HeLa cell lysate and ATP at 37°C for 1 h to allow phosphorylation as previously described (20). An aliquot was collected as a pre-heat inactivation control. The remaining reaction was heat-inactivated at 75°C for 6–8 min. Heat-inactivated samples were then divided into three groups: untreated control, PTP1B-treated, and PTP1B + ZnCl₂-treated. All groups were incubated at 37°C for 1 h. Reactions were terminated by adding 5× SDS sample buffer and heating at 95°C for 5 min. Samples were resolved by SDS-PAGE, and Tarp phosphorylation was assessed by immunoblot using a monoclonal anti-phosphotyrosine antibody. Coomassie staining was used as a loading control.

### Native Tarp dephosphorylation assay

HeLa cells (2 × 10^5^ cells per well in a 6-well plate seeded one day prior to infection) were infected with *Chlamydia trachomatis* serovar L2 at a multiplicity of infection of 100 (MOI = 100) and incubated at 37°C for 2 h. Following infection, media was removed, and cells were scraped and collected in Xenopus buffer (100 mM KCl, 2 mM MgCl_2_, 10 mM HEPES, pH 7.7). Cell lysates were prepared by brief sonication. Lysates were then divided into two groups: one incubated with recombinant PTP1B and the other left untreated. Both reactions were carried out at 37°C for 1 h and terminated by the addition of 5× SDS sample buffer followed by heating at 95°C for 5 min. Samples were resolved by SDS-PAGE, and Tarp phosphorylation was assessed by immunoblotting using a monoclonal anti-phosphotyrosine antibody. Total Tarp levels were detected using a polyclonal anti-Tarp antibody.

### Cell-free type III secretion system (T3SS) induction assay

Cell-free induction of type III secretion system was performed as previously described (54). *C. trachomatis* L2 elementary bodies (EBs), stored at −80°C in sucrose-phosphate-glutamate (SPG) buffer, were thawed on ice. EBs were pelleted by centrifugation at 13,000  RPM for 5 min at 4°C. Supernatants were discarded, and pellets were resuspended in 50  mM potassium acetate buffer (KAC, pH 4.8). EBs were pelleted again under the same conditions, and the supernatant was removed. Pellets were then resuspended in the appropriate experimental solution. Experimental solutions included PTP1B inhibitor (at the indicated concentrations), DMSO (vehicle control at the same concentration as inhibitor), or 3% fetal bovine serum (FBS) as a positive control for T3SS induction. Samples were incubated at 37°C for 30 min. After incubation, EBs were pelleted again at 13,000 RPM for 5 min at 4°C, and the supernatants were transferred to fresh tubes. Pellets were discarded. Supernatants were centrifuged once more at 13,000 RPM for 5 min at 4 °C to remove residual debris. Clarified supernatant was mixed with 5× SDS sample buffer to obtain samples. Samples were loaded onto SDS-PAGE gels for immunoblot analysis. Secreted chlamydial proteins were detected using antibodies against Tarp and MOMP.

### Statistical analysis

All quantitative experiments were performed in at least three independent biological replicates, each with the indicated number of technical replicates. Data are presented as mean + standard deviation (SD), unless otherwise noted. Statistical comparisons between two groups were made using unpaired, two-tailed Student’s *t*-test. For comparisons involving more than two groups, one-way ANOVA followed by Tukey’s post hoc test or Dunnett’s multiple comparison test was applied. *P*-values less than 0.05 were considered statistically significant. Graphing and statistical analyses were conducted using GraphPad Prism version 10.

## SUPPLEMENTAL MATERIAL

Fig. S1 iai.00373-25-s0001.docx Purification of recombinant PTP1B and T3SS induction in response to PTP1B inhibition.

Table S1 iai.00373-25-s0002.xlsx Cloning and SiRNA primers.

ASM does not own the copyrights to Supplemental Material that may be linked to, or accessed through, an article. The authors have granted ASM a non-exclusive, world-wide license to publish the Supplemental Material files. Please contact the corresponding author directly for reuse.

## References

[1] Habtamu E, Harding-Esch EM, Greenland K, Wamyil-Mshelia T, Talero SL, Mishra SK, Lietman TM, Solomon AW, Burton MJ. 2025. Trachoma. Lancet 405:1865–1878. doi:10.1016/S0140-6736(25)00551-3 40412861 PMC7618282

[2] Stelzner K, Vollmuth N, Rudel T. 2023. Intracellular lifestyle of Chlamydia trachomatis and host-pathogen interactions. Nat Rev Microbiol 21:448–462. doi:10.1038/s41579-023-00860-y 36788308

[3] Gitsels A, Sanders N, Vanrompay D. 2019. Chlamydial infection from outside to inside. Front Microbiol 10:2329. doi:10.3389/fmicb.2019.02329 31649655 PMC6795091

[4] Abdelrahman YM, Belland RJ. 2005. The chlamydial developmental cycle. FEMS Microbiol Rev 29:949–959. doi:10.1016/j.femsre.2005.03.002 16043254

[5] Mehlitz A, Rudel T. 2013. Modulation of host signaling and cellular responses by Chlamydia. Cell Commun Signal 11:90. doi:10.1186/1478-811X-11-90 24267514 PMC4222901

[6] Pokrovskaya ID, Szwedo JW, Goodwin A, Lupashina TV, Nagarajan UM, Lupashin VV. 2012. Chlamydia trachomatis hijacks intra-golgi COG complex-dependent vesicle trafficking pathway. Cell Microbiol 14:656–668. doi:10.1111/j.1462-5822.2012.01747.x 22233276 PMC3330190

[7] Keb G, Ferrell J, Scanlon KR, Jewett TJ, Fields KA. 2021. Chlamydia trachomatis TmeA directly activates N-WASP to promote actin polymerization and functions synergistically with TarP during invasion. mBio 12:e02861-20. doi:10.1128/mBio.02861-20 33468693 PMC7845632

[8] Wesolowski J, Paumet F. 2017. Taking control: reorganization of the host cytoskeleton by Chlamydia. F1000Res 6:2058. doi:10.12688/f1000research.12316.1 29225789 PMC5710305

[9] Carabeo RA, Grieshaber SS, Fischer E, Hackstadt T. 2002. Chlamydia trachomatis induces remodeling of the actin cytoskeleton during attachment and entry into HeLa cells. Infect Immun 70:3793–3803. doi:10.1128/IAI.70.7.3793-3803.2002 12065523 PMC128046

[10] Redgrove KA, McLaughlin EA. 2014. The role of the immune response in Chlamydia trachomatis infection of the male genital tract: A double-edged sword. Front Immunol 5:534. doi:10.3389/fimmu.2014.00534 25386180 PMC4209867

[11] Hafner L, Beagley K, Timms P. 2008. Chlamydia trachomatis infection: host immune responses and potential vaccines. Mucosal Immunol 1:116–130. doi:10.1038/mi.2007.19 19079169

[12] Wang X, Wu H, Fang C, Li Z. 2024. Insights into innate immune cell evasion by Chlamydia trachomatis. Front Immunol 15:1289644. doi:10.3389/fimmu.2024.1289644 38333214 PMC10850350

[13] Rucks EA. 2023. Type III secretion in Chlamydia. Microbiol Mol Biol Rev 87:e0003423. doi:10.1128/mmbr.00034-23 37358451 PMC10521360

[14] Ferrell JC, Fields KA. 2016. A working model for the type III secretion mechanism in Chlamydia. Microbes Infect 18:84–92. doi:10.1016/j.micinf.2015.10.006 26515030 PMC4758891

[15] Chen Y-S, Bastidas RJ, Saka HA, Carpenter VK, Richards KL, Plano GV, Valdivia RH. 2014. The Chlamydia trachomatis type III secretion chaperone Slc1 engages multiple early effectors, including TepP, a tyrosine-phosphorylated protein required for the recruitment of CrkI-II to nascent inclusions and innate immune signaling. PLoS Pathog 10:e1003954. doi:10.1371/journal.ppat.1003954 24586162 PMC3930595

[16] Clifton DR, Fields KA, Grieshaber SS, Dooley CA, Fischer ER, Mead DJ, Carabeo RA, Hackstadt T. 2004. A chlamydial type III translocated protein is tyrosine-phosphorylated at the site of entry and associated with recruitment of actin. Proc Natl Acad Sci USA 101:10166–10171. doi:10.1073/pnas.0402829101 15199184 PMC454183

[17] Claywell JE, Matschke LM, Fisher DJ. 2016. The impact of protein phosphorylation on chlamydial physiology. Front Cell Infect Microbiol 6:197. doi:10.3389/fcimb.2016.00197 28066729 PMC5177608

[18] Sah P, Lutter EI. 2020. Hijacking and use of host kinases by chlamydiae. Pathogens 9:1034. doi:10.3390/pathogens9121034 33321710 PMC7763869

[19] Jewett TJ, Fischer ER, Mead DJ, Hackstadt T. 2006. Chlamydial TARP is a bacterial nucleator of actin. Proc Natl Acad Sci USA 103:15599–15604. doi:10.1073/pnas.0603044103 17028176 PMC1622868

[20] Jewett TJ, Dooley CA, Mead DJ, Hackstadt T. 2008. Chlamydia trachomatis tarp is phosphorylated by src family tyrosine kinases. Biochem Biophys Res Commun 371:339–344. doi:10.1016/j.bbrc.2008.04.089 18442471 PMC2394672

[21] Yang S, Zeng J, Yu J, Sun R, Tuo Y, Bai H. 2024. Insights into Chlamydia development and host cells response. Microorganisms 12:1302. doi:10.3390/microorganisms12071302 39065071 PMC11279054

[22] Mital J, Hackstadt T. 2011. Role for the SRC family kinase Fyn in sphingolipid acquisition by chlamydiae. Infect Immun 79:4559–4568. doi:10.1128/IAI.05692-11 21896774 PMC3257913

[23] Claywell JE, Matschke LM, Plunkett KN, Fisher DJ. 2018. Inhibition of the protein phosphatase CppA alters development of Chlamydia trachomatis. J Bacteriol 200:10. doi:10.1128/JB.00419-18 PMC6148467 30038048

[24] Kołodziej-Sobczak D, Sobczak Ł, Łączkowski KZ. 2024. Protein tyrosine phosphatase 1B (PTP1B): a comprehensive review of its role in pathogenesis of human diseases. Int J Mol Sci 25:13. doi:10.3390/ijms25137033 PMC11241624 39000142

[25] Delibegović M, Dall’Angelo S, Dekeryte R. 2024. Protein tyrosine phosphatase 1B in metabolic diseases and drug development. Nat Rev Endocrinol 20:366–378. doi:10.1038/s41574-024-00965-1 38519567

[26] Read NE, Wilson HM. 2024. Recent developments in the role of protein tyrosine phosphatase 1B (PTP1B) as a regulator of immune cell signalling in health and disease. Int J Mol Sci 25:13. doi:10.3390/ijms25137207 PMC11241678 39000313

[27] Sangwan V, Abella J, Lai A, Bertos N, Stuible M, Tremblay ML, Park M. 2011. Protein-tyrosine phosphatase 1B modulates early endosome fusion and trafficking of Met and epidermal growth factor receptors. J Biol Chem 286:45000–45013. doi:10.1074/jbc.M111.270934 22045810 PMC3247994

[28] Monteleone MC, González Wusener AE, Burdisso JE, Conde C, Cáceres A, Arregui CO. 2012. ER-bound protein tyrosine phosphatase PTP1B interacts with Src at the plasma membrane/substrate interface. PLoS One 7:e38948. doi:10.1371/journal.pone.0038948 22701734 PMC3372476

[29] Yue L, Yan M, Tremblay ML, Lin T-J, Li H, Yang T, Song X, Xie T, Xie Z. 2019. PTP1B negatively regulates nitric oxide-mediated Pseudomonas aeruginosa killing by neutrophils. PLoS One 14:e0222753. doi:10.1371/journal.pone.0222753 31532798 PMC6750887

[30] Yue L, Yan M, Chen S, Cao H, Li H, Xie Z. 2020. PTP1B negatively regulates STAT1-independent Pseudomonas aeruginosa killing by macrophages. Biochem Biophys Res Commun 533:296–303. doi:10.1016/j.bbrc.2020.09.032 32958258

[31] Olive AJ, Haff MG, Emanuele MJ, Sack LM, Barker JR, Elledge SJ, Starnbach MN. 2014. Chlamydia trachomatis-induced alterations in the host cell proteome are required for intracellular growth. Cell Host Microbe 15:113–124. doi:10.1016/j.chom.2013.12.009 24439903 PMC3932326

[32] Zadora PK, Chumduri C, Imami K, Berger H, Mi Y, Selbach M, Meyer TF, Gurumurthy RK. 2019. Integrated phosphoproteome and transcriptome analysis reveals Chlamydia-induced epithelial-to-mesenchymal transition in host cells. Cell Rep 26:1286–1302. doi:10.1016/j.celrep.2019.01.006 30699355

[33] Fisher DJ, Adams NE, Maurelli AT. 2015. Phosphoproteomic analysis of the Chlamydia caviae elementary body and reticulate body forms. Microbiology (Reading) 161:1648–1658. doi:10.1099/mic.0.000116 25998263 PMC4681041

[34] Bellomo E, Massarotti A, Hogstrand C, Maret W. 2014. Zinc ions modulate protein tyrosine phosphatase 1B activity. Metallomics 6:1229–1239. doi:10.1039/c4mt00086b 24793162

[35] Jury B, Fleming C, Huston WM, Luu LDW. 2023. Molecular pathogenesis of Chlamydia trachomatis. Front Cell Infect Microbiol 13:1281823. doi:10.3389/fcimb.2023.1281823 37920447 PMC10619736

[36] Bastidas RJ, Elwell CA, Engel JN, Valdivia RH. 2013. Chlamydial intracellular survival strategies. Cold Spring Harb Perspect Med 3:a010256. doi:10.1101/cshperspect.a010256 23637308 PMC3633179

[37] Scidmore MA. 2011. Recent advances in Chlamydia subversion of host cytoskeletal and membrane trafficking pathways. Microbes Infect 13:527–535. doi:10.1016/j.micinf.2011.02.001 21334451 PMC3092832

[38] Saka HA, Valdivia RH. 2010. Acquisition of nutrients by chlamydiae: unique challenges of living in an intracellular compartment. Curr Opin Microbiol 13:4–10. doi:10.1016/j.mib.2009.11.002 20006538 PMC3202608

[39] Thwaites T, Nogueira AT, Campeotto I, Silva AP, Grieshaber SS, Carabeo RA. 2014. The Chlamydia effector TarP mimics the mammalian leucine-aspartic acid motif of paxillin to subvert the focal adhesion kinase during invasion. J Biol Chem 289:30426–30442. doi:10.1074/jbc.M114.604876 25193659 PMC4215226

[40] Zimmerman TJ, Carabeo RA. 2025. Chlamydia trachomatis invasion: a duet of effectors. Biochem Soc Trans 0:363–370. doi:10.1042/BST20240800 40131835 PMC12203932

[41] Rother M, Gonzalez E, Teixeira da Costa AR, Wask L, Gravenstein I, Pardo M, Pietzke M, Gurumurthy RK, Angermann J, Laudeley R, Glage S, Meyer M, Chumduri C, Kempa S, Dinkel K, Unger A, Klebl B, Klos A, Meyer TF. 2018. Combined human genome-wide RNAi and metabolite analyses identify impdh as a host-directed target against Chlamydia infection. Cell Host Microbe 23:661–671. doi:10.1016/j.chom.2018.04.002 29706504

[42] Murphy AM, Sheils OM, McDonald GSA, Kelleher DP. 2005. Detection of a tyrosine phosphatase LAR on intestinal epithelial cells and intraepithelial lymphocytes in the human duodenum. Mediators Inflamm 2005:23–30. doi:10.1155/MI.2005.23 15770063 PMC1513056

[43] Boivin B, Chaudhary F, Dickinson BC, Haque A, Pero SC, Chang CJ, Tonks NK. 2013. Receptor protein-tyrosine phosphatase α regulates focal adhesion kinase phosphorylation and ErbB2 oncoprotein-mediated mammary epithelial cell motility. J Biol Chem 288:36926–36935. doi:10.1074/jbc.M113.527564 24217252 PMC3873551

[44] Samet JM, Silbajoris R, Wu W, Graves LM. 1999. Tyrosine phosphatases as targets in metal-induced signaling in human airway epithelial cells. Am J Respir Cell Mol Biol 21:357–364. doi:10.1165/ajrcmb.21.3.3656 10460753

[45] Liu R, Mathieu C, Berthelet J, Zhang W, Dupret J-M, Rodrigues Lima F. 2022. Human protein tyrosine phosphatase 1B (PTP1B): from structure to clinical inhibitor perspectives. IJMS 23:7027. doi:10.3390/ijms23137027 35806030 PMC9266911

[46] Krishnan N, Konidaris KF, Gasser G, Tonks NK. 2018. A potent, selective, and orally bioavailable inhibitor of the protein-tyrosine phosphatase PTP1B improves insulin and leptin signaling in animal models. J Biol Chem 293:1517–1525. doi:10.1074/jbc.C117.819110 29217773 PMC5798283

[47] Agrawal N, Dhakrey P, Pathak S. 2023. A comprehensive review on the research progress of PTP1B inhibitors as antidiabetics. Chem Biol Drug Des 102:921–938. doi:10.1111/cbdd.14275 37232059

[48] Liu Z, Gao H, Zhao Z, Huang M, Wang S, Zhan J. 2023. Status of research on natural protein tyrosine phosphatase 1B inhibitors as potential antidiabetic agents: update. Biomedicine & Pharmacotherapy 157:113990. doi:10.1016/j.biopha.2022.113990 36459712

[49] Kumar A, Rana D, Rana R, Bhatia R. 2020. Protein tyrosine phosphatase (PTP1B): a promising drug target against life-threatening ailments. Curr Mol Pharmacol 13:17–30. doi:10.2174/1874467212666190724150723 31339082

[50] Olloquequi J, Cano A, Sanchez-López E, Carrasco M, Verdaguer E, Fortuna A, Folch J, Bulló M, Auladell C, Camins A, Ettcheto M. 2022. Protein tyrosine phosphatase 1B (PTP1B) as a potential therapeutic target for neurological disorders. Biomed Pharmacother 155:113709. doi:10.1016/j.biopha.2022.113709 36126456

[51] Vieira MNN, Lyra E Silva NM, Ferreira ST, De Felice FG. 2017. Protein tyrosine phosphatase 1B (PTP1B): a potential target for alzheimer’s therapy? Front Aging Neurosci 9:7. doi:10.3389/fnagi.2017.00007 28197094 PMC5281585

[52] Scidmore MA. 2005. Cultivation and laboratory maintenance of Chlamydia trachomatis. Curr Protoc Microbiol Chapter 11:Unit doi:10.1002/9780471729259.mc11a01s00 18770550

[53] Carabeo RA, Dooley CA, Grieshaber SS, Hackstadt T. 2007. Rac interacts with Abi-1 and WAVE2 to promote an Arp2/3-dependent actin recruitment during chlamydial invasion. Cell Microbiol 9:2278–2288. doi:10.1111/j.1462-5822.2007.00958.x 17501982

[54] Jamison WP, Hackstadt T. 2008. Induction of type III secretion by cell-free Chlamydia trachomatis elementary bodies. Microb Pathog 45:435–440. doi:10.1016/j.micpath.2008.10.002 18984037 PMC2592499

