# Supplementary material for: Protein tyrosine phosphatase PTP1B is a positive regulator of the intracellular development of Chlamydia trachomatis
Source: Infect Immun. 2025 Oct 20;93(11):e00373-25. doi: 10.1128/iai.00373-25 (PMC12604487; doi:10.1128/iai.00373-25)
Supplement: Fig. S1 — Purification of recombinant PTP1B and T3SS induction in response to PTP1B inhibition. [file iai.00373-25-s0001.docx]

**
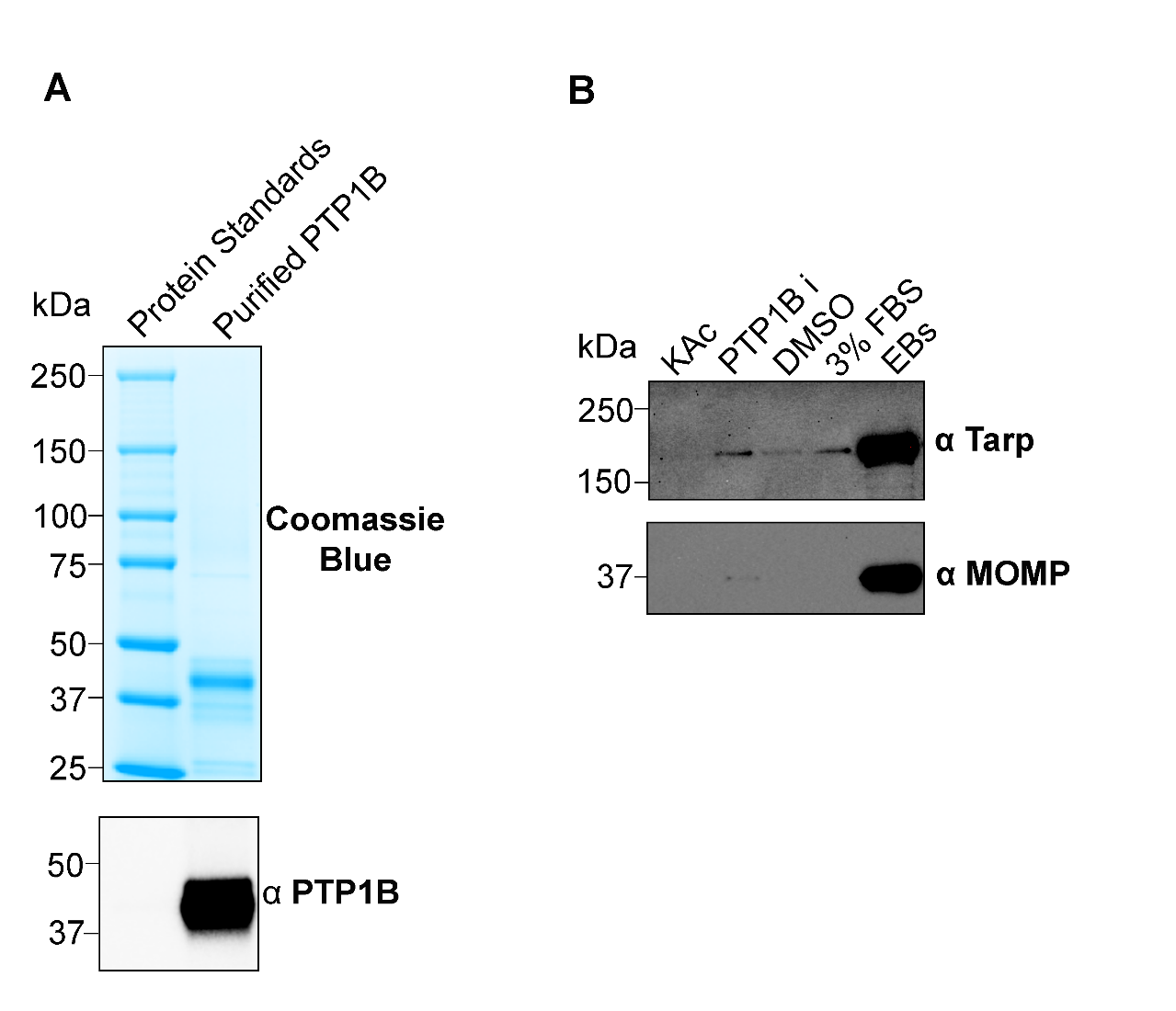
**

**Supplemental Figure S1. Purification of recombinant PTP1B and T3SS induction in response to PTP1B inhibition**.

**(A)** Recombinant PTP1B was expressed in E. coli, purified by affinity chromatography, and analyzed by SDS-PAGE. Coomassie blue staining (top) and immunoblotting with PTP1B-specific antibodies (bottom) confirmed protein purity and identity. Molecular mass markers are shown in kilodaltons (kDa **(B)** Induction of type III secretion system (T3SS) activity in a cell-free system upon treatment with PTP1B inhibitor. C. trachomatis EBs were incubated in potassium acetate (KAc) buffer with PTP1B inhibitor or DMSO. 3% FBS was used as a positive control. *C. trachomatis* elementary bodies (EBs) were loaded in the last lane as additional control. Supernatants were collected and analyzed by immunoblotting with anti-Tarp and anti-MOMP antibodies to assess effector secretion.
